# Supplementary figures and images for: High-throughput micro-CT analysis identifies sex-dependent biomarkers of erosive arthritis in TNF-Tg mice and differential response to anti-TNF therapy
Source: PLoS One. 2024 Jul 5;19(7):e0305623. doi: 10.1371/journal.pone.0305623 (PMC11226038; doi:10.1371/journal.pone.0305623)

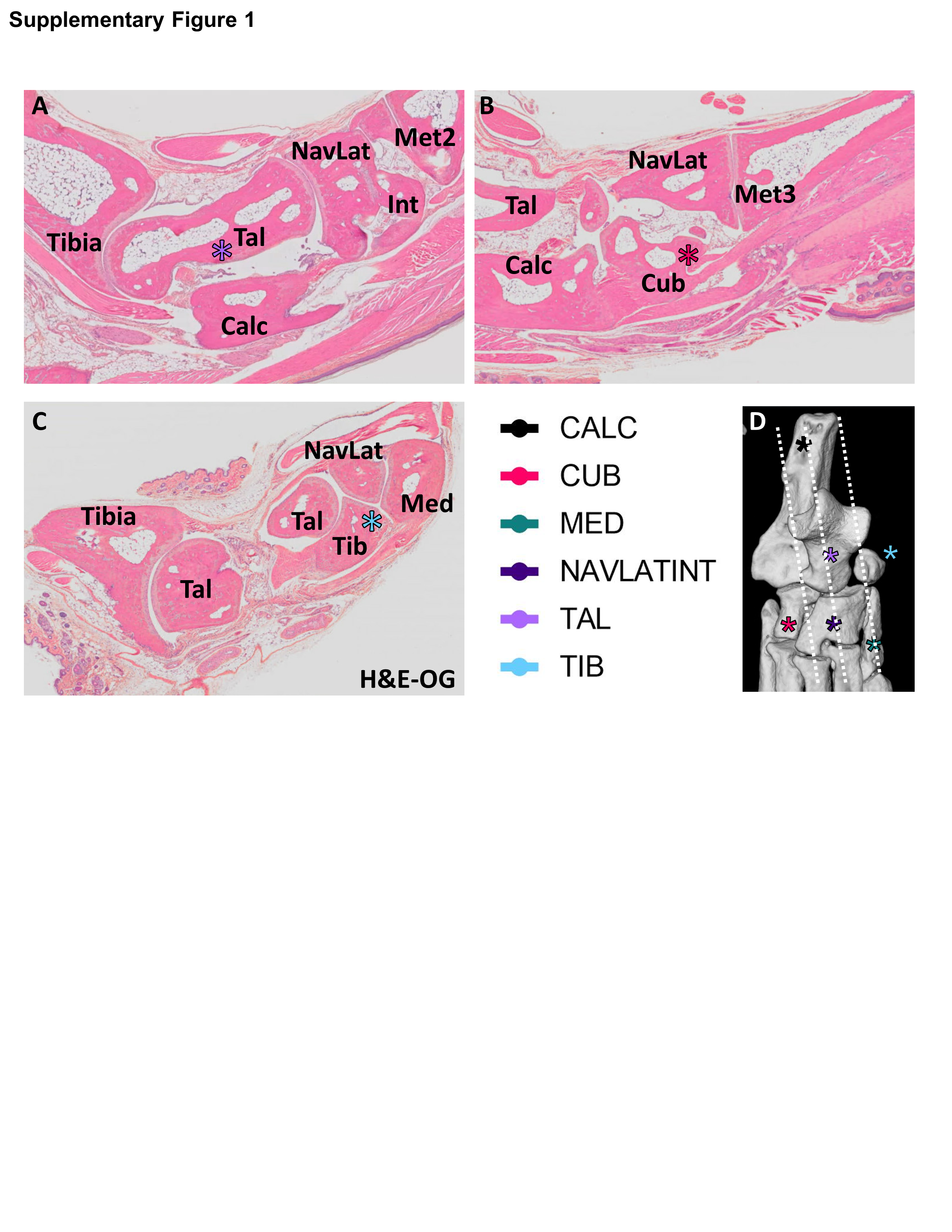

Supplement: S1 Fig — Representative H&E-OG-stained histologic sections of the tarsal region in a wild-type ankle is provided to demonstrate the identification of the talus (A, purple asterisk), cuboid (B, pink asterisk), and tibiale (C, blue asterisk) relative to closely articulating bones. A micro-CT image is provided with specific tarsal bones highlighted by color coded asterisks (D, reproduced from Fig 4A) to directly visualize the sectioning planes (white dashed lines) utilized to evaluate these particular bones by histology. (TIF) [file pone.0305623.s001.TIF]

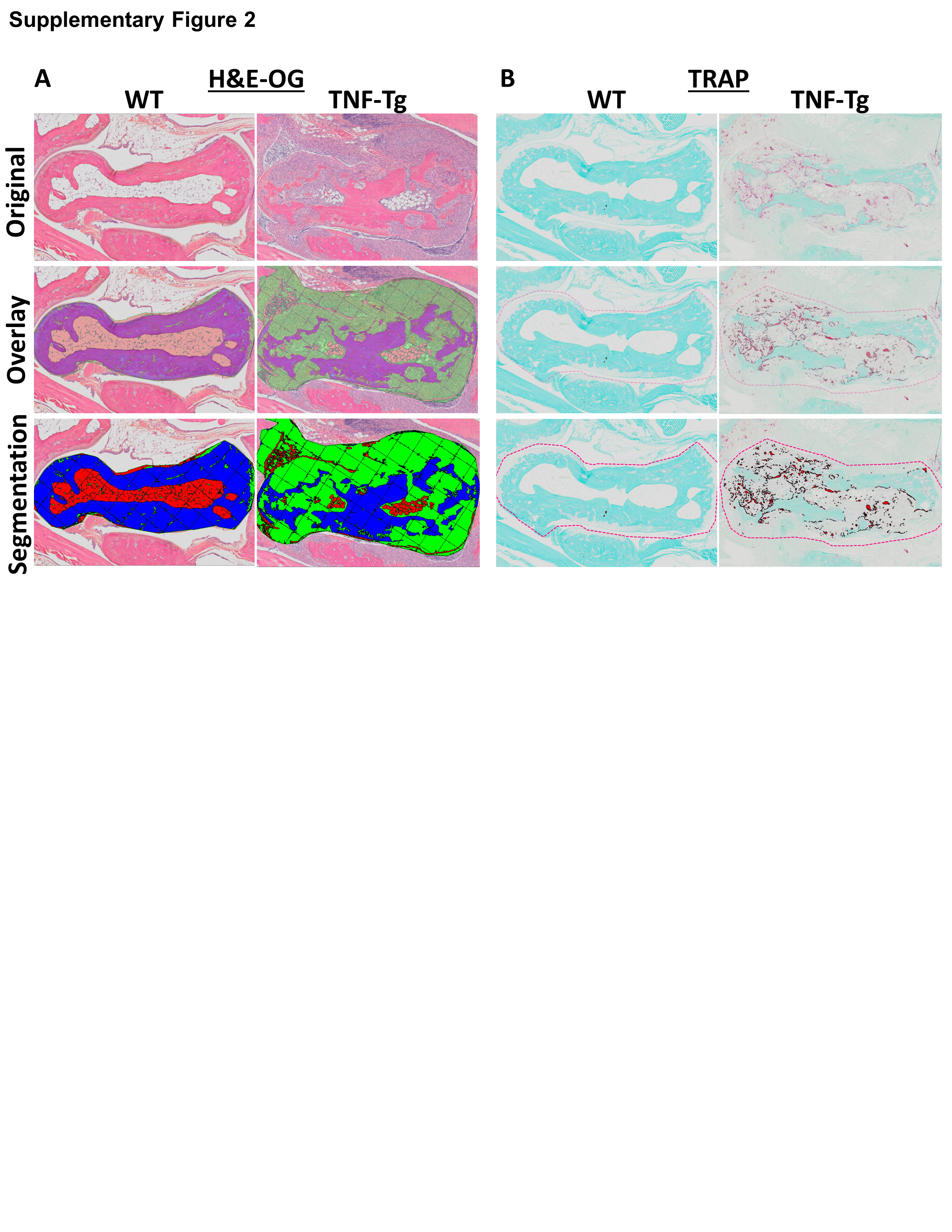

Supplement: S2 Fig — Representative histologic sections of a talus stained with H&E-OG (A) and TRAP (B) from WT (left) and TNF-Tg (right) cohorts with demonstration of segmentation for quantification of corresponding areas using the Visiopharm software are provided. For the H&E-OG, the original staining is shown (top) with the resultant segmentation (bottom; green = synovium, blue = bone and soft tissue, red = adipose and background) adjacent to a corresponding transparent overlay of the staining and segmentation (middle) (A). Similarly, the TRAP staining (blue = bone and soft tissue, red = TRAP) is segmented with a bright red overlay, and the pink dashed line represents the region of interest surrounding the talus where the analysis was performed (B). (TIF) [file pone.0305623.s002.TIF]

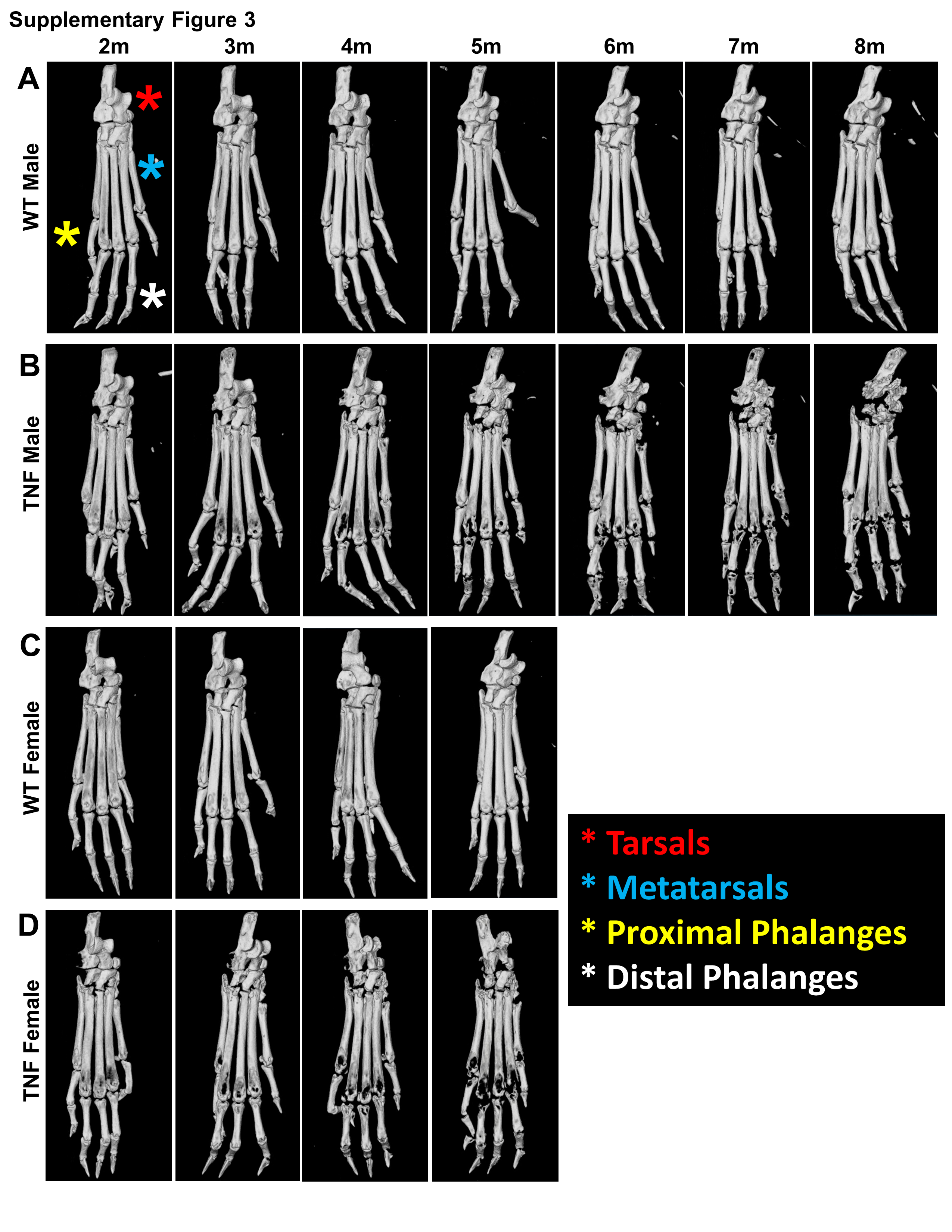

Supplement: S3 Fig — A representative 3D rendering of micro-CT datasets for WT (A, tarsals = red asterisk, metatarsals = blue asterisk, proximal phalanges = yellow asterisk, distal phalanges = white asterisk) and TNF-Tg (B) male dorsal hindpaws from 2–8 months of age at monthly intervals are provided. Similar images for WT (C) and TNF-Tg (D) female dorsal hindpaws from 2–5 months of age are shown. (TIF) [file pone.0305623.s003.TIF]

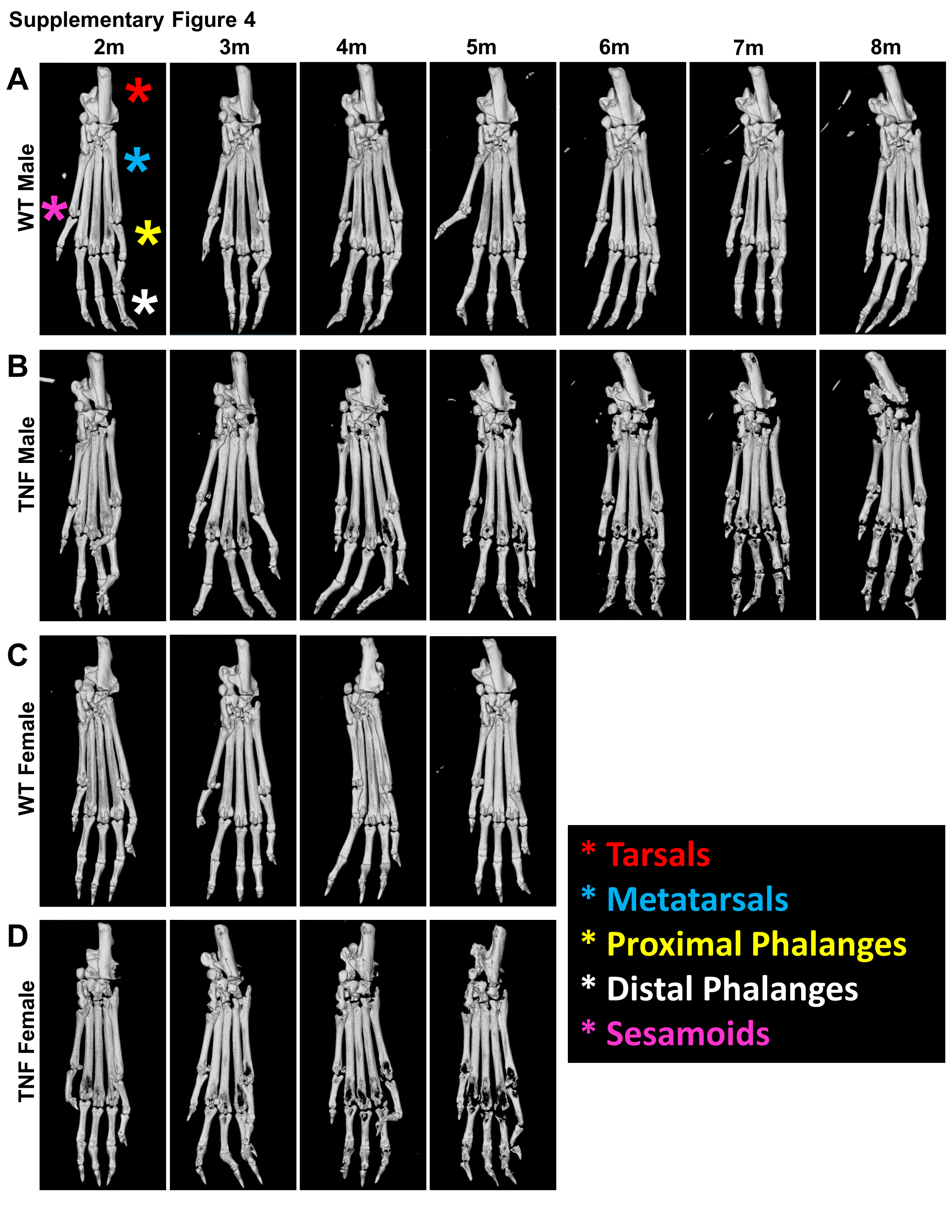

Supplement: S4 Fig — A representative 3D rendering of micro-CT datasets for WT (A, tarsals = red asterisk, metatarsals = blue asterisk, proximal phalanges = yellow asterisk, distal phalanges = white asterisk, sesamoids = pink asterisk) and TNF-Tg (B) male plantar hindpaws from 2–8 months of age at monthly intervals are provided. Similar images for WT (C) and TNF-Tg (D) female plantar hindpaws from 2–5 months of age are shown. (TIF) [file pone.0305623.s004.TIF]

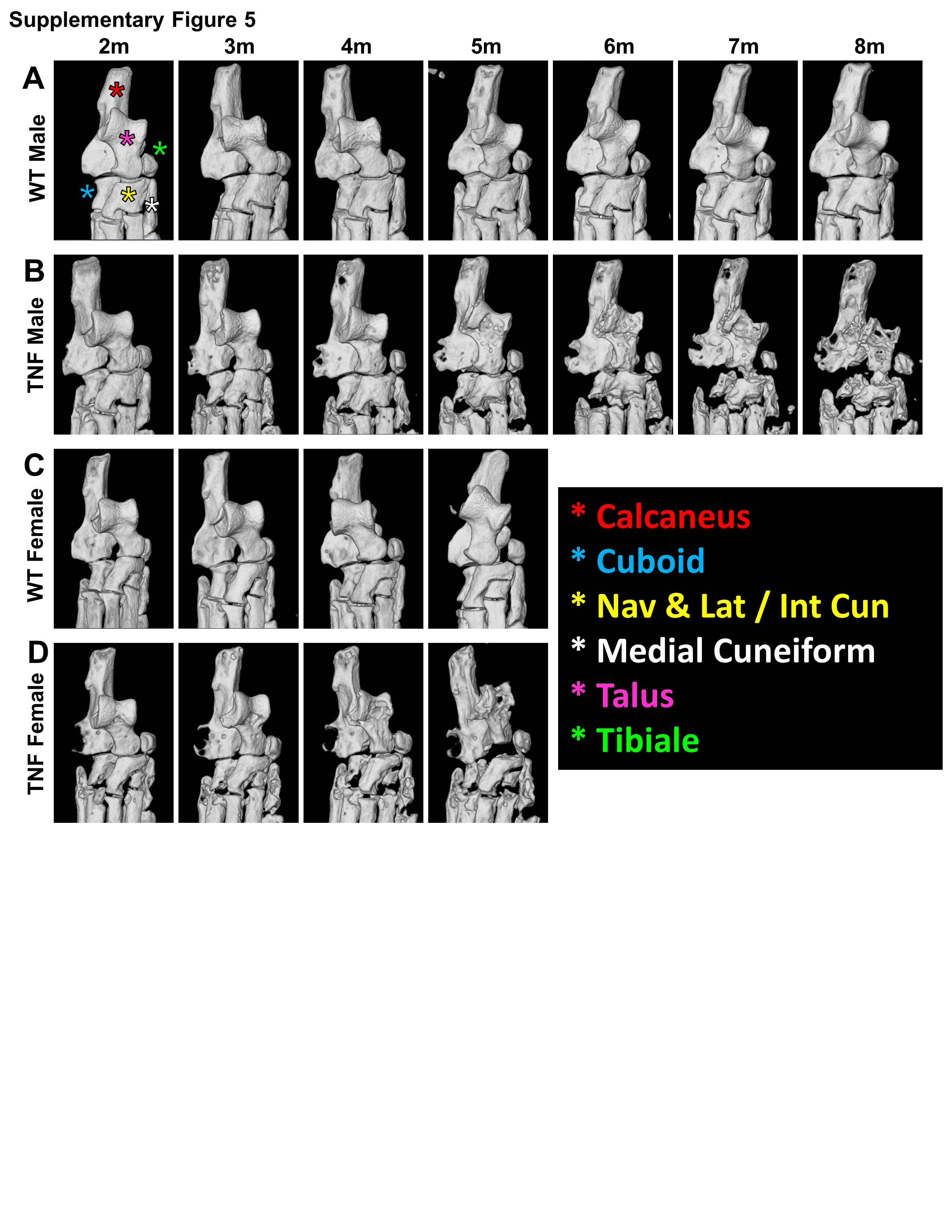

Supplement: S5 Fig — A representative 3D rendering of micro-CT datasets for WT (A, calcaneus = red asterisk, cuboid = blue asterisk, navicular & lateral cuneiform / intermediate cuneiform [variably fused] = yellow asterisk, medial cuneiform = white asterisk, talus = pink asterisk, and tibiale = green asterisk) and TNF-Tg (B) male tarsal regions from 2–8 months of age at monthly intervals are provided. Similar images for WT (C) and TNF-Tg (D) female tarsal compartments from 2–5 months of age are shown. (TIF) [file pone.0305623.s005.TIF]

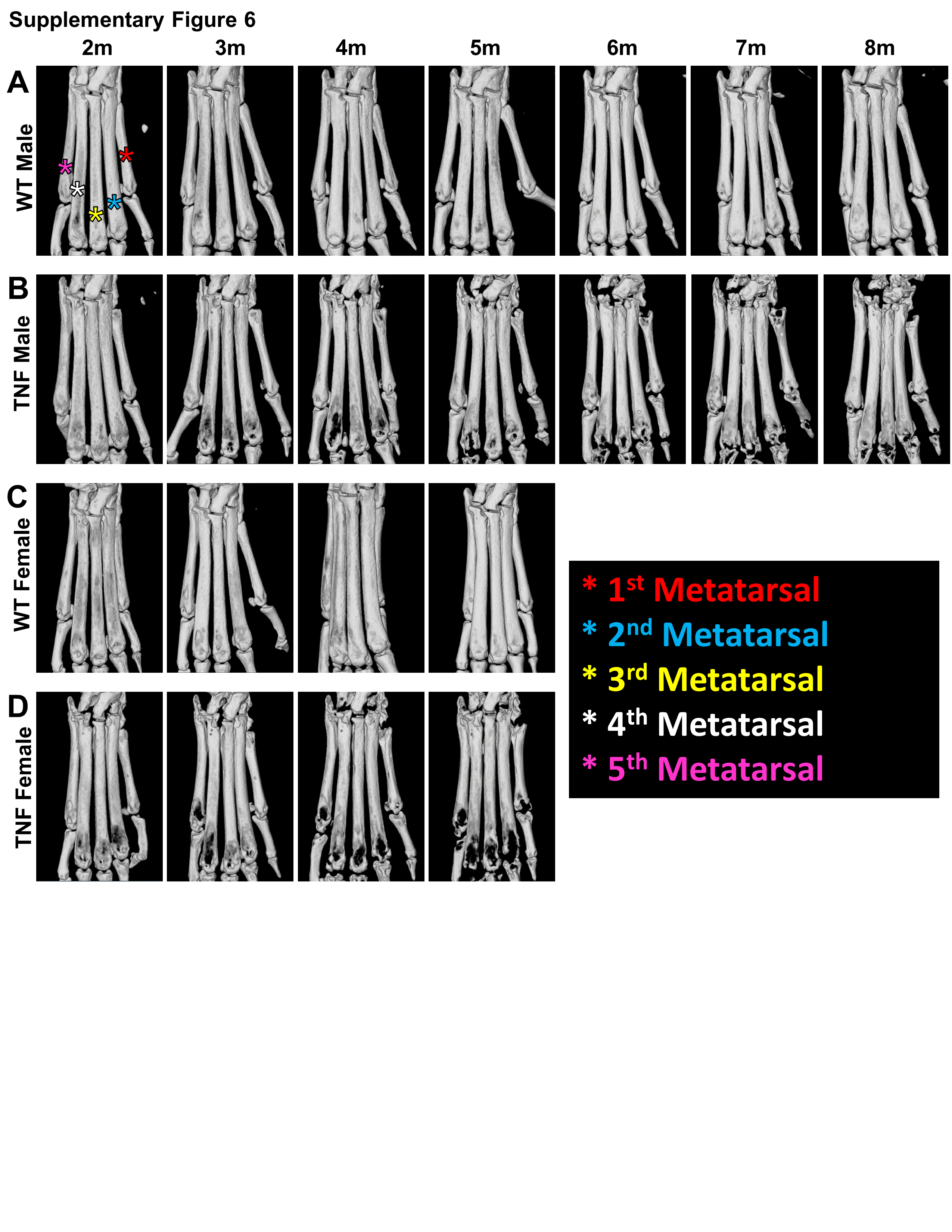

Supplement: S6 Fig — A representative 3D rendering of micro-CT datasets for WT (A, 1st metatarsal = red asterisk, 2nd metatarsal = blue asterisk, 3rd metatarsal = yellow asterisk, 4th metatarsal = white asterisk, 5th metatarsal = pink asterisk) and TNF-Tg (B) male metatarsal regions from 2–8 months of age at monthly intervals are provided. Similar images for WT (C) and TNF-Tg (D) female metatarsal compartments from 2–5 months of age are shown. (TIF) [file pone.0305623.s006.TIF]

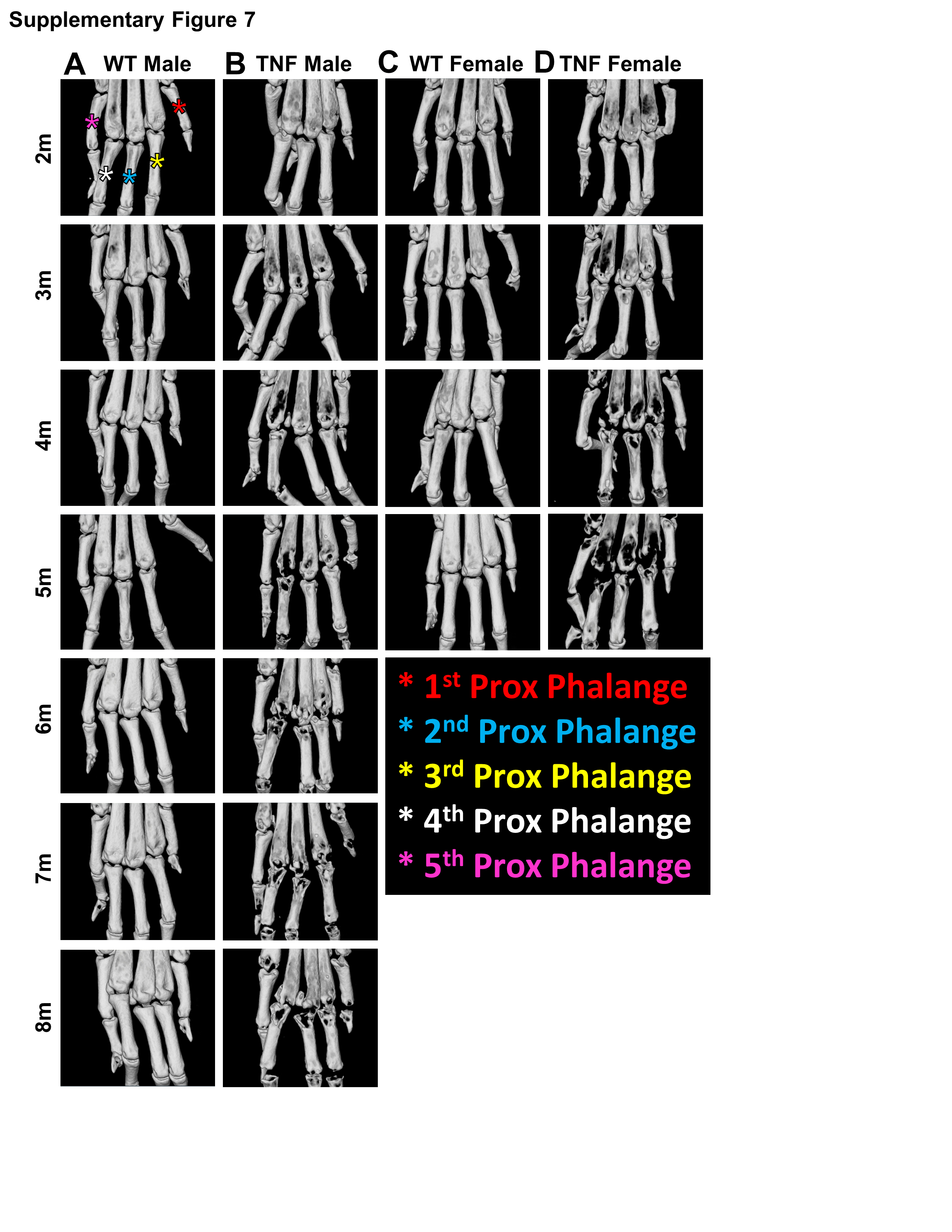

Supplement: S7 Fig — A representative 3D rendering of micro-CT datasets for WT (A, 1st proximal phalange = red asterisk, 2nd proximal phalange = blue asterisk, 3rd proximal phalange = yellow asterisk, 4th proximal phalange = white asterisk, 5th proximal phalange = pink asterisk) and TNF-Tg (B) male proximal phalange regions from 2–8 months of age at monthly intervals are provided. Similar images for WT (C) and TNF-Tg (D) female proximal phalange compartments from 2–5 months of age are shown. Bones are numbered 1–5 based on digits from medial to lateral. (TIF) [file pone.0305623.s007.TIF]

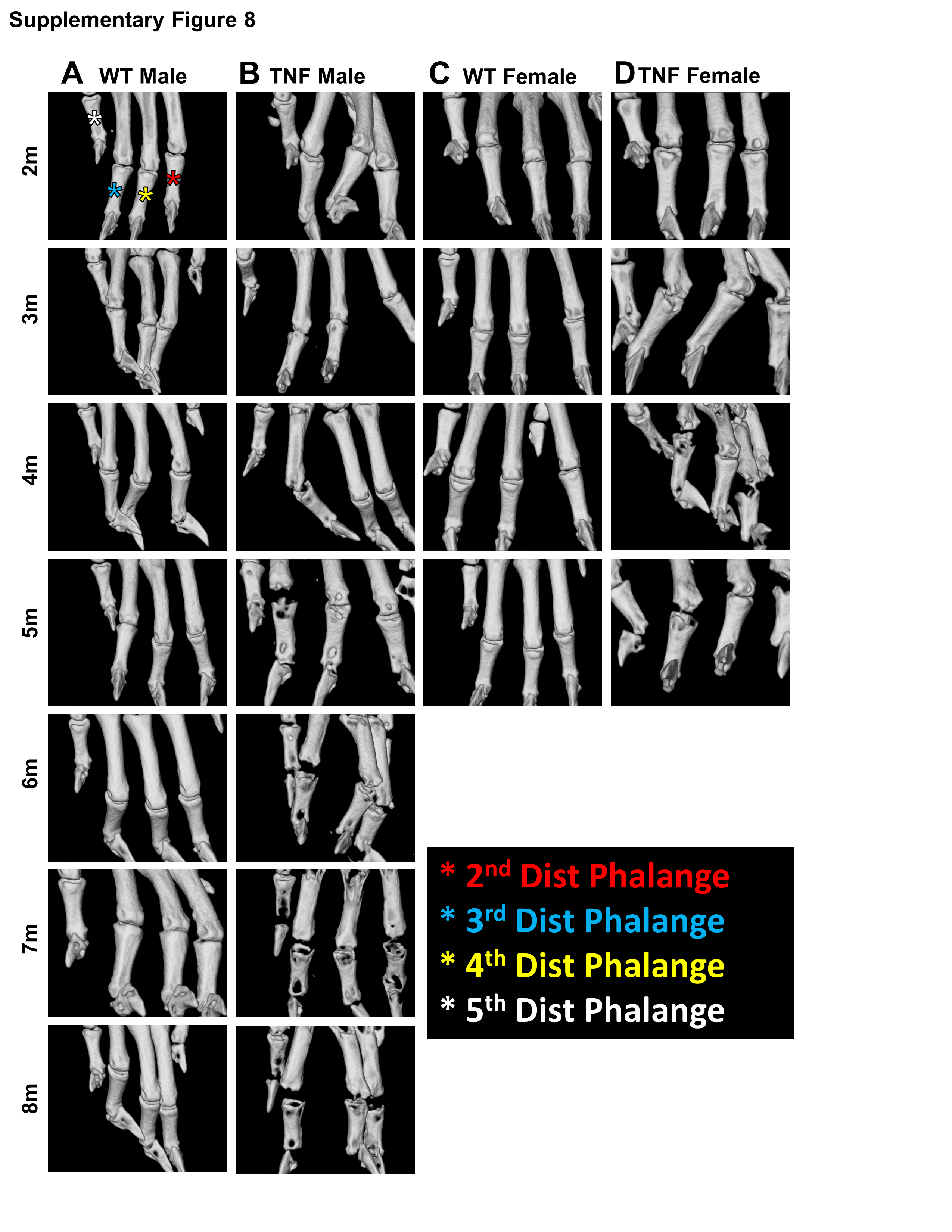

Supplement: S8 Fig — A representative 3D rendering of micro-CT datasets for WT (A, 2nd distal phalange = red asterisk, 3rd distal phalange = blue asterisk, 4th distal phalange = yellow asterisk, 5th distal phalange = white asterisk) and TNF-Tg (B) male distal phalange regions from 2–8 months of age at monthly intervals are provided. Similar images for WT (C) and TNF-Tg (D) female distal phalange compartments from 2–5 months of age are shown. Bones are numbered 2–5 based on digits from medial to lateral; note the 1st digit does not have a distal phalange. (TIF) [file pone.0305623.s008.TIF]

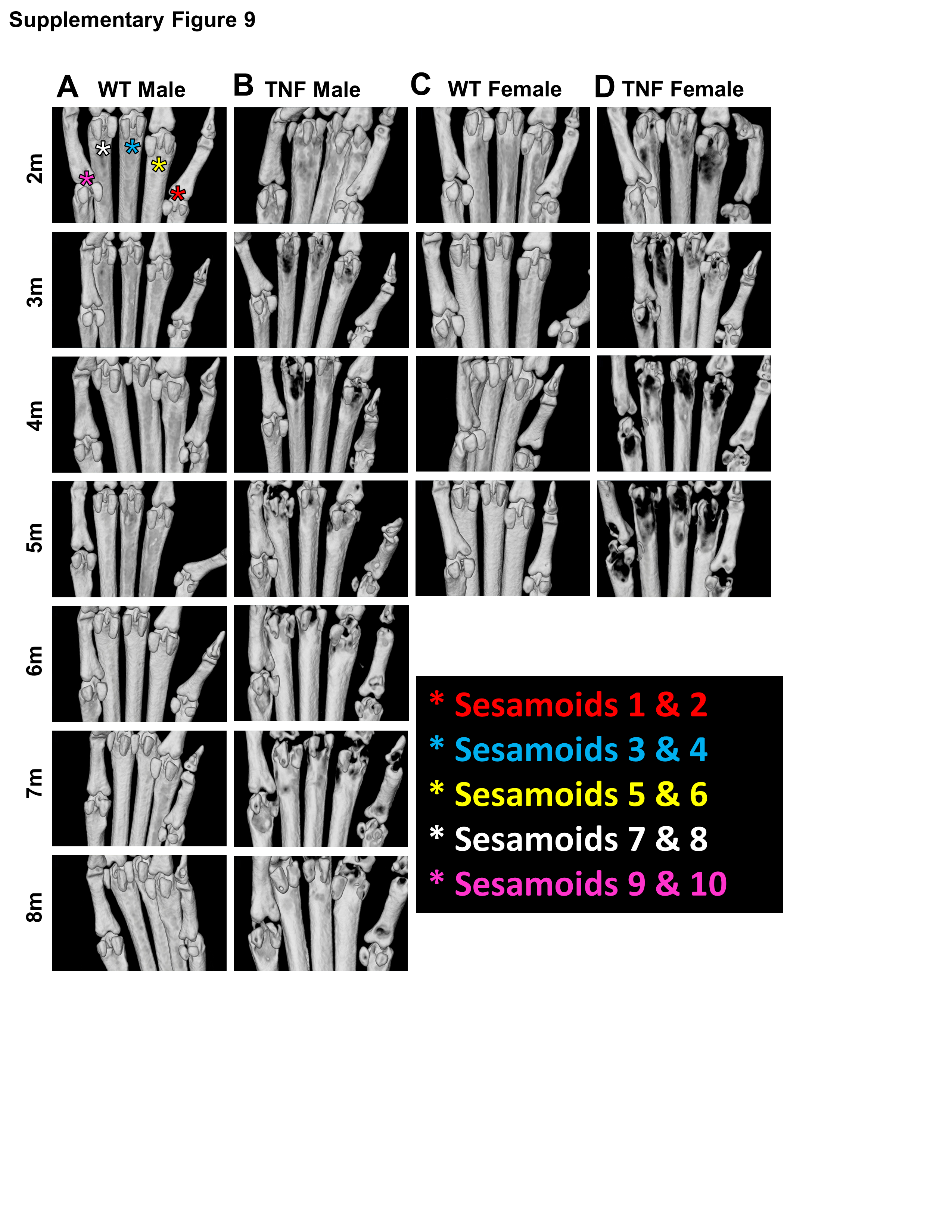

Supplement: S9 Fig — A representative 3D rendering of micro-CT datasets for WT (A, sesamoids 1 & 2 = red asterisk, sesamoids 3 & 4 = blue asterisk, sesamoids 5 & 6 = yellow asterisk, sesamoids 7 & 8 = white asterisk, sesamoids 9 & 10 = pink asterisk) and TNF-Tg (B) male sesamoid regions from 2–8 months of age at monthly intervals are provided. Similar images for WT (C) and TNF-Tg (D) female sesamoid compartments from 2–5 months of age are shown. Bones are numbered 1–10 from medial to lateral with 2 sesamoids per digit. (TIF) [file pone.0305623.s009.TIF]

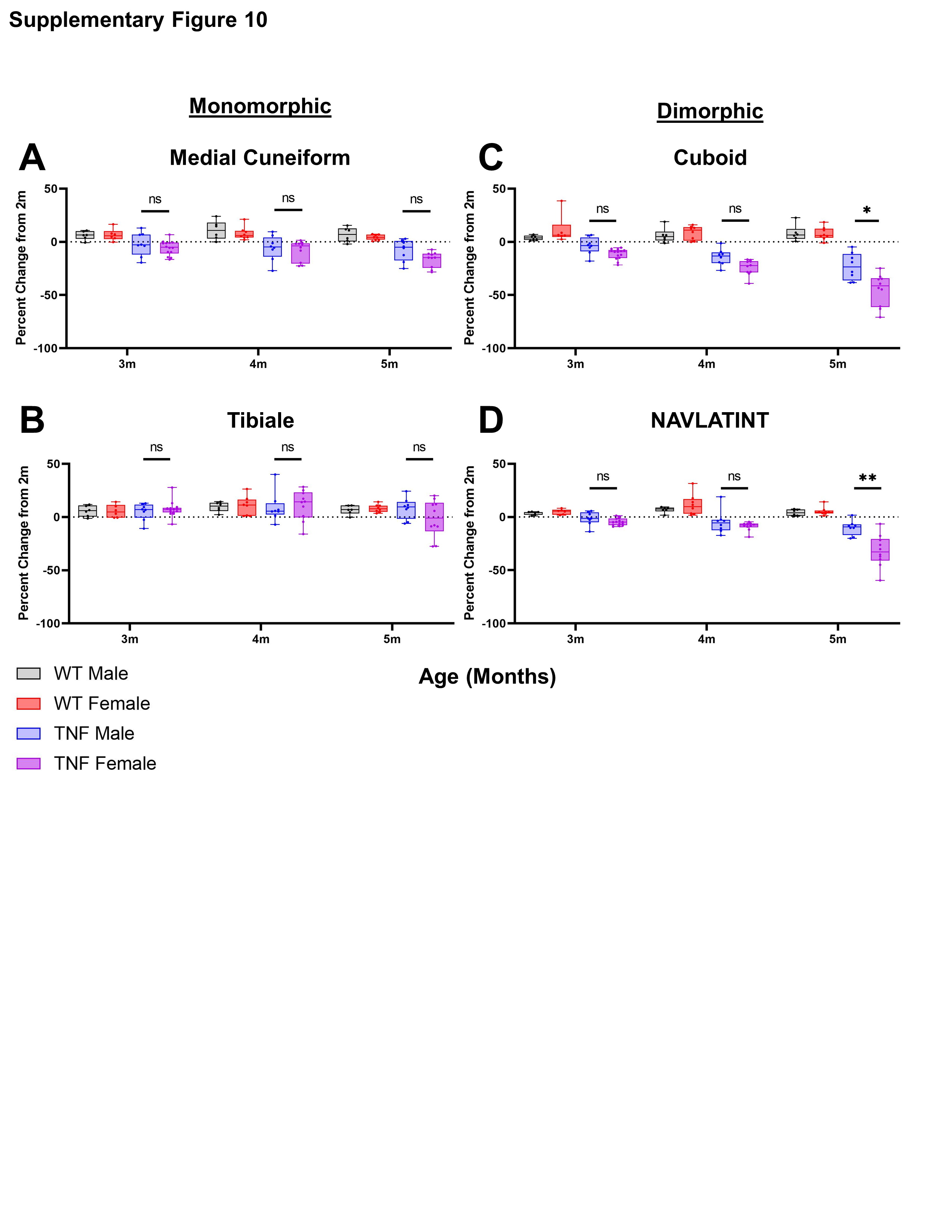

Supplement: S10 Fig — Corresponding with the sexually monomorphic (yellow asterisks) and dimorphic (red asterisks) bones highlighted in Fig 5O and 5P, quantification of normalized bone volumes across time for the medial cuneiform (A) and tibiale (B) (monomorphic) along with the cuboid (C) and NAVLATINT (D) (dimorphic) are provided. Statistics: Mixed effects analysis with Tukey’s multiple comparisons (A-D); * p<0.05, ** p<0.01. (TIF) [file pone.0305623.s010.TIF]

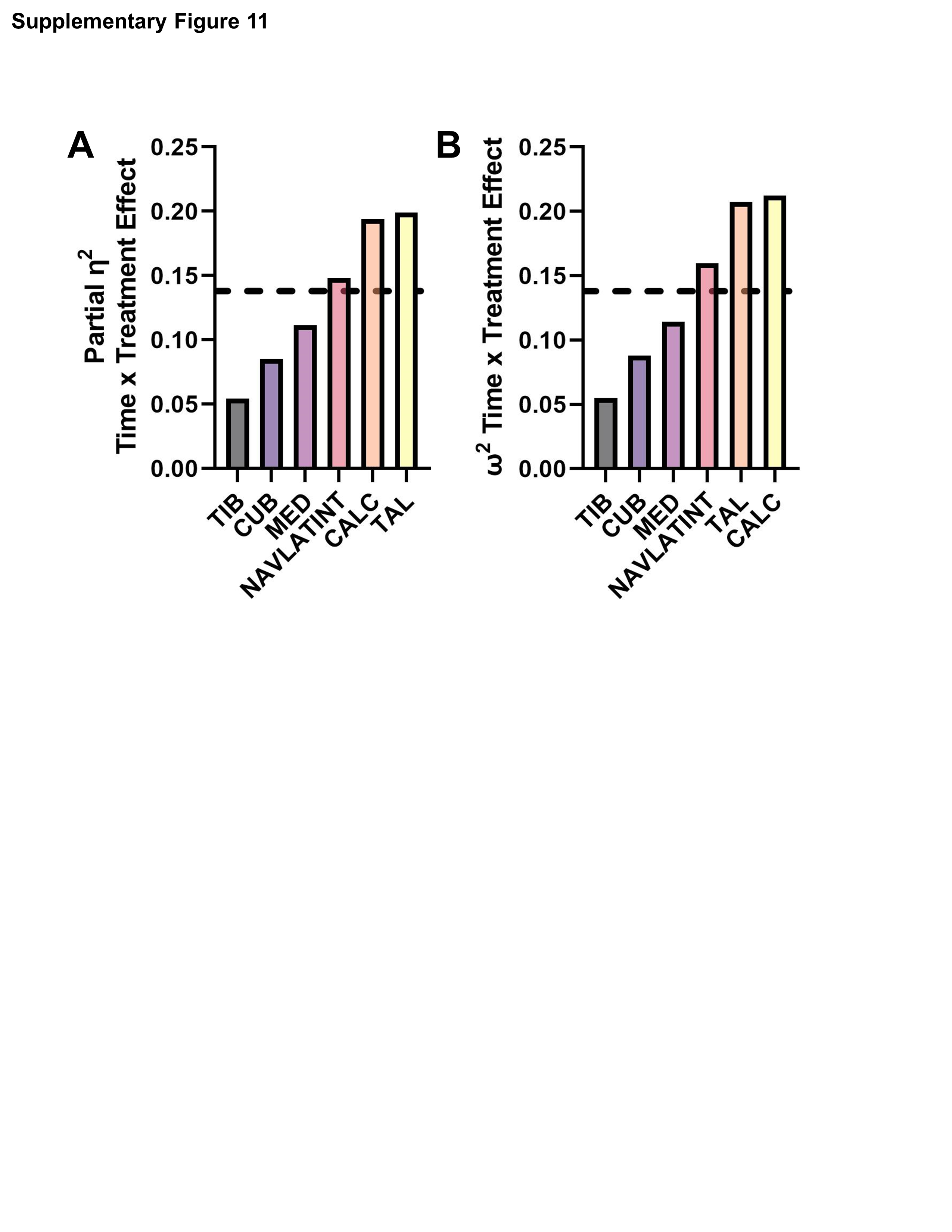

Supplement: S11 Fig — Associated with the quantification of effect size by eta-squared (η2) shown in Fig 6K, we further evaluated whether the same relationships remained consistent across alternative methods for effect size assessment, including partial η2 (A) and omega-squared (ω2) (B) (equations provided in Materials and methods, Statistics). Consistent with evaluation of η2 both partial η2 and ω2 identified the talus (partial η2 = 0.20, ω2 = 0.21) and calcaneus (partial η2 = 0.19, ω2 = 0.21) as the bones with the greatest effect sizes (large effect size >0.138, dashed black lines). (TIF) [file pone.0305623.s011.TIF]
